# Clonal strains of the fresh-market potato cultivar Russet Norkotah changed the domestication gene *CDF1*

**DOI:** 10.1093/plphys/kiaf321

**Published:** 2025-08-13

**Authors:** Kirk Richard Amundson, Maria Isabel Vales, Isabelle Jocelyn DeMarco, Weier Guo, Isabelle Marie Henry, Luca Comai

**Affiliations:** Department of Plant Biology and Genome Center, University of California Davis, Davis, CA 95616, USA; Department of Horticultural Sciences, Texas A&M University, College Station, TX 77843, USA; Department of Plant Biology and Genome Center, University of California Davis, Davis, CA 95616, USA; Department of Plant Biology and Genome Center, University of California Davis, Davis, CA 95616, USA; Department of Plant Biology and Genome Center, University of California Davis, Davis, CA 95616, USA; Department of Plant Biology and Genome Center, University of California Davis, Davis, CA 95616, USA

Dear Editor,

Clonal selection is an established breeding method for further improvement of varieties selected from sexual crosses (Foster and Aranzana 2018). Variants displaying favorable traits can be found serendipitously or identified from large screens. In some cases, the causal mutations have been elucidated and range from chromosomal rearrangements to point mutations (Azuma et al. 2009; Tan et al. 2019; Vondras et al. 2019). Here, we report the characterization of clonal variants of a tetraploid potato cultivar and the molecular mechanisms underlying their superior field behavior.

In 1987, the North Dakota Agricultural Experiment Station and USDA-ARS released the variety Russet Norkotah (RN) (Fig. 1A), selected from a cross of two experimental genotypes (Johansen et al. 1988). RN initiates tuberization early in the season. Due to its high yield and good tuber quality, RN became very popular. Subsequently, clonal selection was carried out to improve plant vigor, yield, and adaptation to lower latitudes (Miller et al. 1999). Breeders identified clonal variants as larger plants defined as “giant hills” or “bolters” (Carson and Howard 1944; Hawkes 1946) among thousands of RN clonal individuals. Further evaluation of hundreds of separate clonal strains yielded several improved clones that initially became widely grown in Colorado and Texas and later elsewhere in the nation. The Texas clonal strains TXNS112, TXNS223, TXNS278, and TXNS296 displayed later maturity, higher vigor, and yield than RN (Miller et al. 1999; Jansky and Miller 2010), as well as milder Verticillium wilt symptoms (Jansky and Miller 2010). Based on a 12-year evaluation at two Texas locations, the total yield and marketable yield of the TXNS# were not significantly different from RN; however, in 4 of these 12 years, the clonal variants significantly out-yielded RN (Fig. 1B). RN and its strains represent the second most grown potato cultivar group in the United States after Russet Burbank (a processing potato). TXNS296 and TXNS278 are the most popular strains (Jennings 2022). The genetic or epigenetic changes responsible for the TXNS# phenotypes are unknown (Pandey et al. 2021).

**Figure 1.**
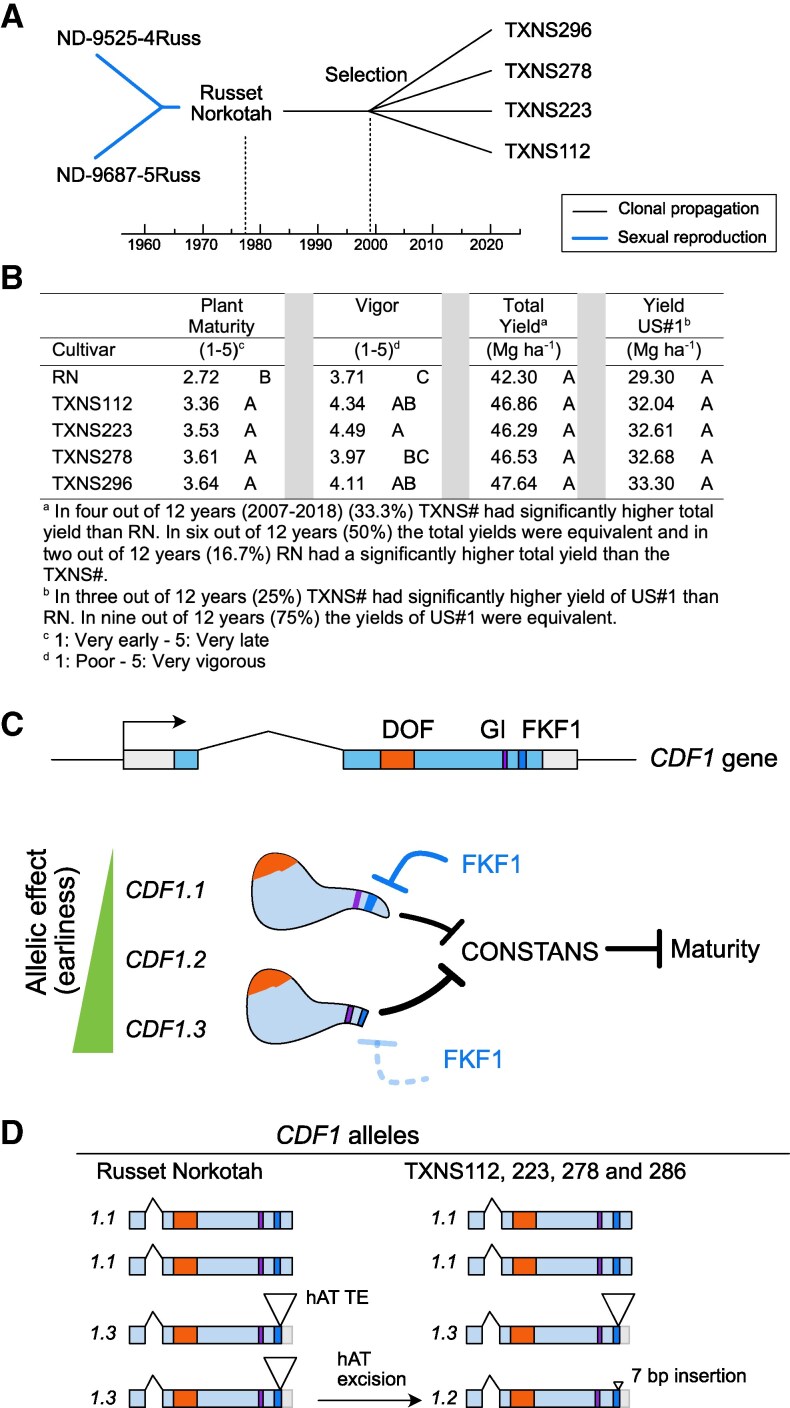
Characterization of Russet Norkotah and its Texas strains. **A)** History of Russet Norkotah and some Texas clonal strains (TXNS#). Genotypes TXNS278 and 296 represented ∼60% of the Russet Norkotah 2021 US acreage. **B)** Phenotypic trait values (least square means) measured from 2007 to 2018 in two Texas locations (Springlake and Dalhart). The letters indicate clones that do not differ significantly at *P* = 0.05. In the case of yield traits, no significant differences were observed between RN and TXNS# due to genotype-by-year interactions; TXNS# had significantly higher yields than RN in >25% of the years. **C)**  *CDF1* gene, protein, mechanism of action, and allelic strength. DOF: domain of unknown function; GI: GIGANTEA binding site; FKF1: FLAVIN-BINDING, KELCH REPEAT, F-BOX-binding site. **D)** Genotype of RN and TXNS# strains at *CDF1* locus.

To identify candidate changes, we sequenced the four popular Texas clonal strains of RN (TXNS#), obtaining 30–50× coverage of Illumina reads, which we mapped to the DM1-3 v6.1 genome (Pham et al. 2020) and compared to publicly available RN reads (Pham et al. 2017). Single nucleotide polymorphism (SNP) allele dosage analysis confirmed that all four variants were RN clones (Supplementary Fig. S1). Dosage plots failed to reveal large-scale chromosomal variants relative to RN (Supplementary Fig. S2). Mutation analysis (Supplementary Methods) identified high-impact mutations shared by all four strains in three genes: SoltuDM01G006960, which encodes a DUF2828 domain-containing protein, SoltuDM05G005140 (*CYCLING DOF FACTOR1* (*CDF1*)), and SoltuDM07G017380, which encodes a RAD16 homolog that contributes to UV tolerance in Arabidopsis (Alrayes et al. 2023). The potential roles of RAD16 and DUF2828 will require additional investigations.

There is much information, however, on *CDF1* (Imaizumi et al. 2005; Kloosterman et al. 2013; Salaria et al. 2020; Hoopes et al. 2022). Specifically, the giant hill trait (Yarwood 1946), and significant differences in plant maturity and vigor between RN and TXNS# are consistent with the characterization of *CDF1* as a pleiotropic regulatory locus in seed plants and a domestication gene involved in photoperiod-modulated tuberization and maturity in potatoes. Its full-length product (encoded by allele *CDF1.1*) suppresses the expression of flowering activator *CONSTANS* and has been associated with delayed maturity, a trait unfavorable at high latitudes (Imaizumi et al. 2005; Kloosterman et al. 2013; Salaria et al. 2020; Hoopes et al. 2022). In addition, *CDF1* modulates tuberization onset by negative regulation of *ORESARA1* (Shi et al. 2024). Many modern potato varieties have alleles encoding truncated proteins, either because of a transposon insertion (*CDF1.3*), or because of a 7-bp insertion scar presumably left by the transposon excision (*CDF1.2*, *CDF1.4*). These truncated forms lose negative regulation by FLAVIN-BINDING, KELCH REPEAT, F-BOX (FKF1) and result in earlier maturity (Imaizumi et al. 2005; Caraza-Harter and Endelman 2022; Hoopes et al. 2022). Their action, however, is not equivalent. *CDF1.2* has properties intermediate between late-maturing *CDF1.1* and early-maturing *CDF1.3* (Caraza-Harter and Endelman 2022; Hoopes et al. 2022). The difference between *CDF1.2* and *CDF1.3* may be due to their differential response to the repressive action of antisense RNA. Some *CDF1*-like genes are negatively regulated by the convergent transcription of a long noncoding RNA called *FLORE* (Henriques et al. 2017). If *FLORE* is involved in *CDF1* regulation, excision of the *CDF1.3 TE* may strengthen its effect. Further, heterozygosity of multiple varieties at this locus suggests trait modulation through dosage effects (Caraza-Harter and Endelman 2022; Hoopes et al. 2022).

The relaxed mutation search (3+ positive reads) we employed is predicted to result in a high false discovery rate (Henry et al. 2014). Focusing on *CDF1*, we examined read alignment to the locus (Supplementary Fig. S3 and Supplementary Table S1). In tetraploid RN, we identified heterozygosity for two alleles, each present in duplex dosage (Figs. 1 and 2 and Supplementary Fig. S3). One allele type corresponded to *CDF1.3*, which resulted from the insertion of a hAT transposon in the second exon of *CDF1* (Fig. 2A and Supplementary Table S4). The second allele resembled *CDF1.1* but displayed a 3-bp deletion whose consequences are unknown, but given the diversity of *CDF1.1* alleles, it is likely wild type (Caraza-Harter and Endelman 2022; Hoopes et al. 2022). While the RN genotype is *CDF1.1/CDF 1.1/CDF1.3/CDF1.3*, we reliably detected a previously absent allele, *CDF1.2*, in all four strains (TXNS#). The presence in this allele of a 7-bp insertion part of a direct repeat is consistent with the excision of the hAT transposon from *CDF1.3*, resulting in *CDF1.1/CDF 1.1/CDF1.2/CDF1.3* genotype (Fig. 1).

**Figure 2.**
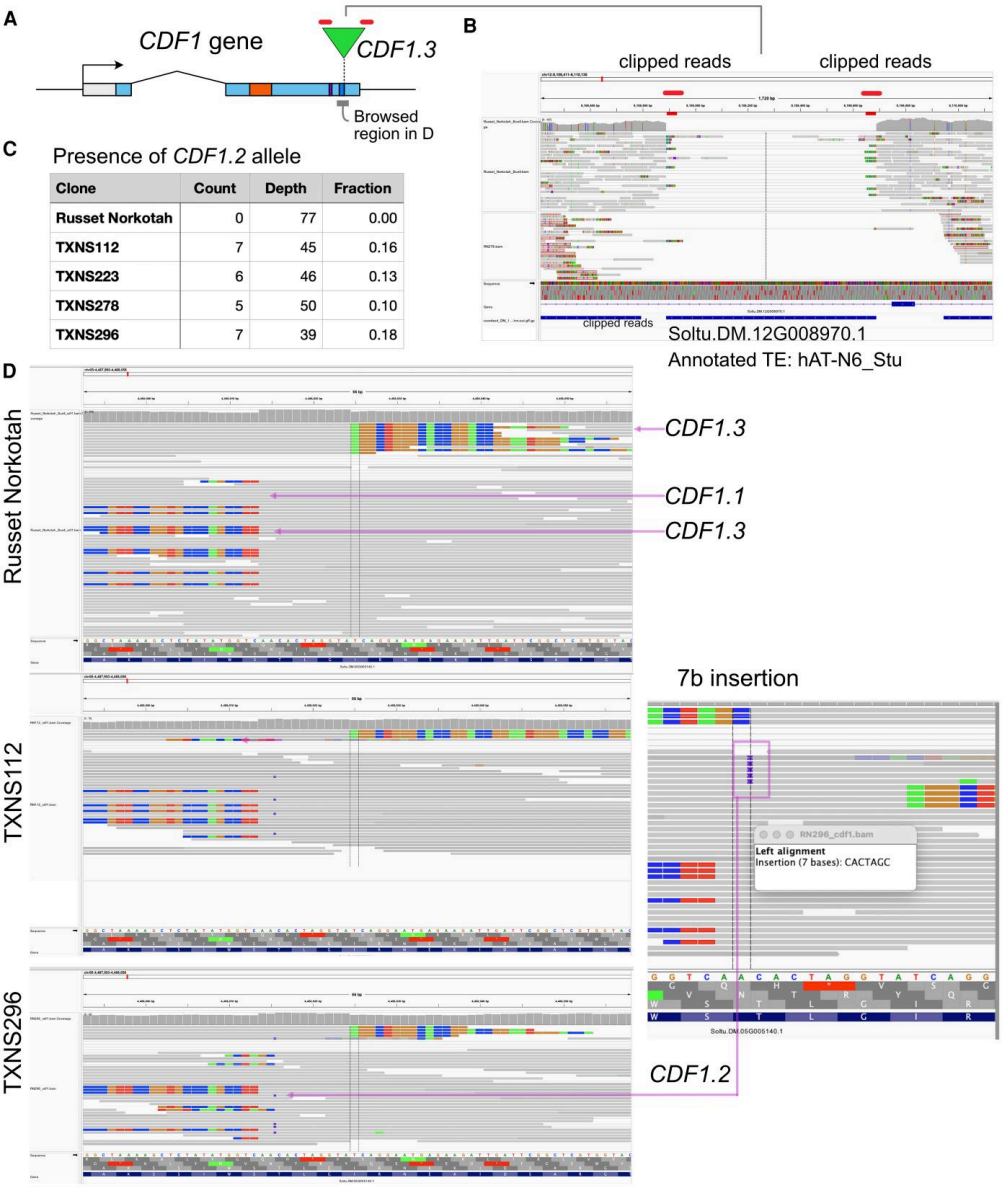
Identification and characterization of a novel allele in TXNS# cultivars. **A)** Structure of the CDF1 gene with location of the transposon inserted in CDF1.3. **B)** Identification of the transposon element's flanks inserted inside the CDF1.3 allele. We extracted the longest soft-clipped parts of the CDF1.3 reads (from both the left and right) and aligned them to the potato reference genome (DM1.3 v6.1 from SpudDB) using BLAST. An exemplary pair of hits at the 5′ and 3′ ends of a hAT are illustrated. This particular element is 800 bp long and is inserted in gene Soltu.DM.12G008970. The lower track displays TE annotation from the SpudDB. It identifies the element as hAT_N6_Stu. **C)** Scoring of CDF1.2-specific reads. The expected variant allele frequency for a simplex (1/4) mutation is 0.25. The DNA used for the sequencing libraries was isolated from leaves, which are formed by L1 (epidermis), L2 (mesophyll and parenchyma), and L3 (vascular system). **D)** Comparison of RN and RN 112 reads alignment in the IGV browser (see Supplementary Fig. S3 for the other clones). Methods for library construction are described in Supplementary Methods. Relevant to the uniqueness of read-reference alignment, at all tested mismatch rates, the mappability score (see Supplementary Methods) across the entire CDF1 locus was 1, ruling out complicating effects of read mappability.

The establishment of a somatic mutation in clonal crops involves the invasion of one of three layers, L1, L2, and L3, depending on the stem cell in which the mutation originated (Satina et al. 1940). As a result, the frequency of a somatic mutant allele in a tetraploid such as potato is less than the expected 0.25 and proportional to the fraction of mutant cells in the chimera. We assigned sequence reads to alleles by their alignment pattern and used their count to derive the variant allele frequency (VAF) for allele *CDF1.2* (Fig. 2B). The observed VAF was lower than the expected 0.25 per allele and, therefore, did not match expectations of uniform presence in all cells, suggesting chimerism. Our analysis of other potato mutants indicates that L1 mutations sampled in the leaf of a periclinal chimera have VAF = ∼0.06 (Amundson et al. 2023). The L2 is thought to form the largest fraction of cells in a leaf. Uniformity of L2 and L3 has been observed in different chimeras and is thought to result from layer invasion (Thompson and Olmo 1963; Howard et al. 1964; Howard 1972). Mutations present in L2 and L3 have VAF = ∼0.2 (Amundson et al. 2023). The observed VAF of the *CDF1.2* allele (Fig. 2) was consistent with chimerism and suggested that the mutation affected the L2 or jointly the L2 and L3.

Excision of the transposon could modify expression of the CDF1.2 allele. We used available RNAseq reads (Levy et al. 2018) to determine allelic contribution to expression. In leaves of RN, CDF1 expressed at low level with the CDF1.1 two alleles representing ∼3/4 of the reads (Supplementary Table S2). In leaves of TXNS278, the CDF1.1 alleles expressed similarly. CDF1.2 was detected at the expected, low level. The read coverage of the gene was low but sufficient to rule out major regulatory changes. More subtle modulation, however, may occur.

Our finding that the selection of all four tested TXNS# clones is associated with the appearance of the *CDF1.2* allele, later maturity, higher vigor, and increased yield does not prove causality. Other mutations were found in the clones, uniquely or shared (Supplementary Tables S3–S5). Given that *CDF1.2* confers maturity intermediate between *CDF1.1* and *CDF1.3* (Caraza-Harter and Endelman 2022; Hoopes et al. 2022), the formation of this allele in four independent clones is strongly suggestive of causality and consistent with the hypothesis that excision of the hAT transposon from the *CDF1.3* allele influenced traits through modulation of CDF1 function.

What further analysis would confirm this hypothesis? Several approaches are possible and are listed in order of increasing complexity. A first approach may be to screen new “giant hill” variants of RN or varieties with the *CDF1.3* allele to further associate the appearance of the *CDF1.2* allele to the trait. A second approach would be to introduce the *CDF1.2* allele as a transgene. A third class of approaches recreate or generate novel CDF1 alleles with targeted editing. For example, the CDF1.3 TE could be excised, or edited at its ends to inhibit transposase-mediated excision; alternatively, edits targeting the promoter or antisense RNA could generate novel regulatory alleles (Rodríguez-Leal et al. 2017; Wan et al. 2025). These approaches may also be instrumental in the breeding of improved clones. However, approaches requiring tissue culture regeneration carry the potential to mobilize TEs (Hirochika 1993 ), destabilize the genome (Fossi et al. 2019), and eliminate layer-specific mutations through the dissociation of periclinal chimeras (Amundson et al. 2023).

In summary, we have shown that the four improved RN clones (TXNS#) evaluated have later maturity, increased vigor, and equal or higher yield than the original RN. These improvements are associated with the excision of a transposon from the *CDF1.3* allele and production of known intermediate maturity allele *CDF1.2*. An active transposon and the interaction of multiple alleles at one major domestication locus (*CDF1*) may thus contribute to the plasticity and agronomic performance of this polyploid clonal crop and, specifically, in the success of the second most popular potato variety in the United States.

## Supplementary Material

kiaf321_Supplementary_Data

## Data Availability

The data underlying this article are available in the NCBI SRA repository at https://www.ncbi.nlm.nih.gov/sra/PRJNA1241303, and can be accessed with accession number PRJNA1241303.

## Supplementary data

The following materials are available in the online version of this article.

**Supplementary Figure S1.** Phylogenetic analysis of experimental lines and a representative potato panel using ∼6,000 loci and carried out according to Amundson et al. (2023), Am. J. Potat. Res. The TXNS# is positioned on the RN phylogenetic branch (blue highlight). Part of the total tree is omitted.

**Supplementary Figure S2.** Dosage plots of Russet Norkotah and its Texas TXNS# strains. For dosage analysis, read depth is binned in 100-kb intervals and plotted along each chromosomal axis. In the euchromatic chromosomal arms, the Solacap reads have an average depth of 60. The TXNS# reads of 40. The highly variable regions are typically centromeric and pericentromeric.

**Supplementary Figure S3.** Short genomic read alignment to the second exons of the *CDF1* locus.

**Supplementary Figure S4.** Short RNAseq read alignment to the *CDF1* locus (top) and to the second exon’s region (bottom) containing the 7-bp duplication (position 4,888,016 of chr.5) of allele *CDF1.2*. Most reads display the G, G, and T SNPs and correspond to alleles *CDF1.1.*

**Supplementary Table S1.** Variants in *CDF1* gene coding sequence. Variants in second exon were dense enough to phase by visualizing short read alignment on the IGV browser. For Russet Norkotah, the four chromosomes display two unique haplotypes. The TE excision in the TXNS# occurred on either hap1 or hap2 and in repulsion with 3-bp deletion haplotype.

**Supplementary Table S2.** Expression of CDF1 alleles. RNAseq reads were inspected between nucleotide 4,487,950 and 4,488,020 for distinguishing polymorphism to determine their allelic origin. *CDF1.2* was identified by the presence of a 7-bp duplication at position 4,488,016 of chr.5. Notably, RN tissue is homogeneous for the allele genotype, while it is likely that TXNS278 is a periclinal chimera (see the text). This would dilute the *CDF1.2* allele proportionally. RN: Russet Norkotah original clone. TXNS278: clonal variant of RN.

**Supplementary Table S3.** RN high-confidence mutations (RN_high_confidence_mutations_v2.tsv). Stringently filtered of mutations in RN and its clones. 10.6084/m9.figshare.29298572.

**Supplementary Table S4.** RN lower stringency mutations (RN_rough_mutations_v2.tsv). Standardly filtered mutations in RN and its clones. 10.6084/m9.figshare.29298572.

**Supplementary Table S5.** SNPeff analysis of mutations (filt2_q20_rn_calls_v2_snpeff.vcf.gz). 10.6084/m9.figshare.29298572.

## Dive Curated Terms

The following phenotypic, genotypic, and functional terms are of significance to the work described in this paper:

FKF1 Gramene: AT1G68050
FKF1 Araport: AT1G68050

GI Gramene: AT1G22770
GI Araport: AT1G22770

CDF1 Gramene: AT5G62430
CDF1 Araport: AT5G62430
