# Supplementary material for: Clonal strains of the fresh-market potato cultivar Russet Norkotah changed the domestication gene CDF1
Source: Plant Physiol. 2025 Aug 13;198(4):kiaf321. doi: 10.1093/plphys/kiaf321 (PMC12344491; doi:10.1093/plphys/kiaf321)
Supplement: kiaf321_Supplementary_Data [file kiaf321_supplementary_data.zip › Amundson_supplemental_figures.docx]

**Clonal strains of the fresh-market potato cultivar Russet Norkotah changed the domestication gene CDF1**

Kirk Amundson^1,2^ , M. Isabel Vales^3^, Isabelle J. DeMarco^1^, Weier Guo^1^, Isabelle M. Henry^1^, and Luca Comai^1^,*

^1^Department of Plant Biology and Genome Center, University of California Davis, Davis, CA 95616

^2^Current address: Department of Biology, University of Massachusetts Amherst, Amherst, MA 01003

^3^Department of Horticultural Sciences, Texas A&M University, College Station, TX 77843

**Supplemental Figures**


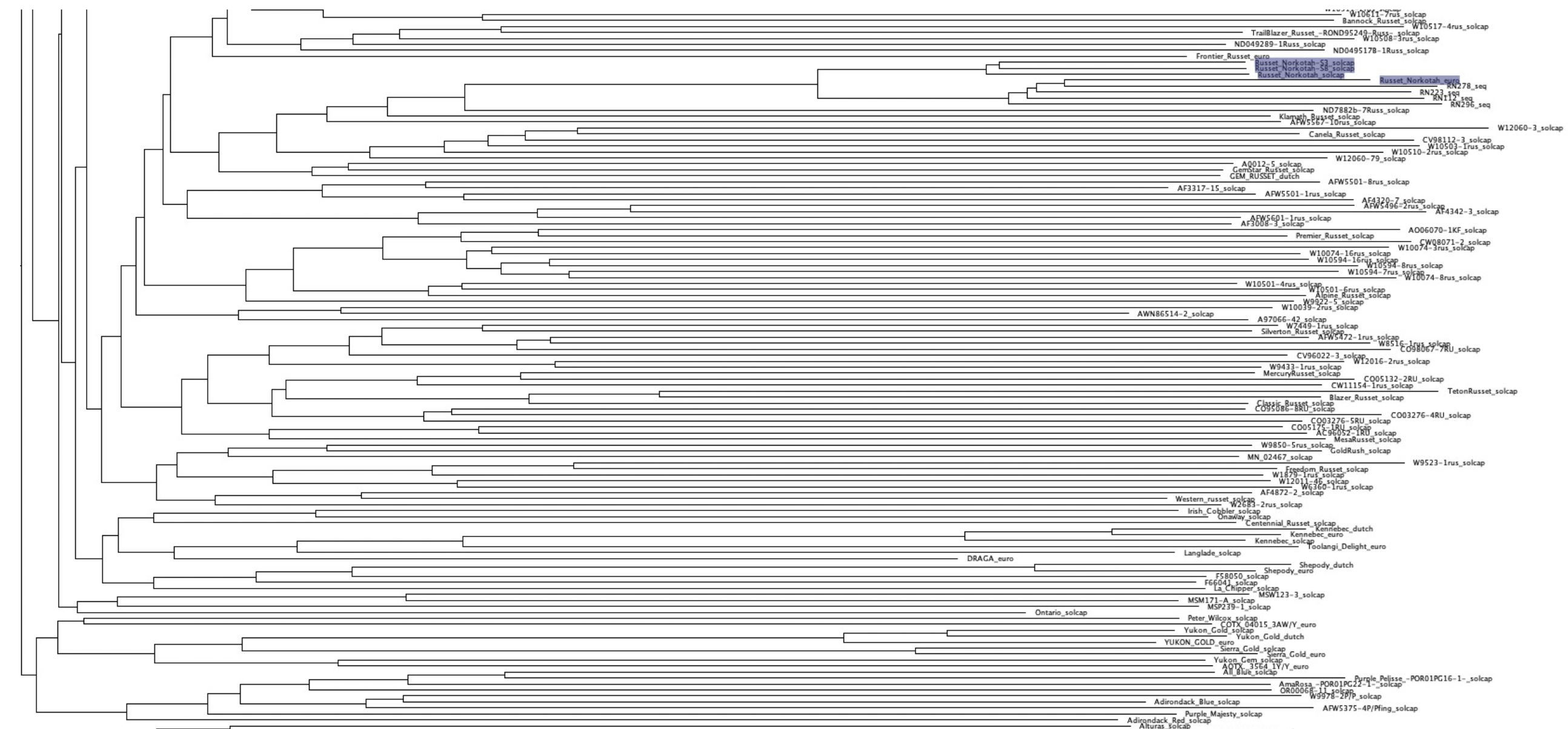


**Supplementary Figure 1**. Phylogenetic analysis of experimental lines and a representative potato panel using ~6000 loci and carried out according to Amundson et al. (2023) Am. J. Potat. Res. The TXNS# are positioned on the RN phylogenetic branch (blue highlight). Part of the total tree is omitted.

Russet Norkotah Solcap

| 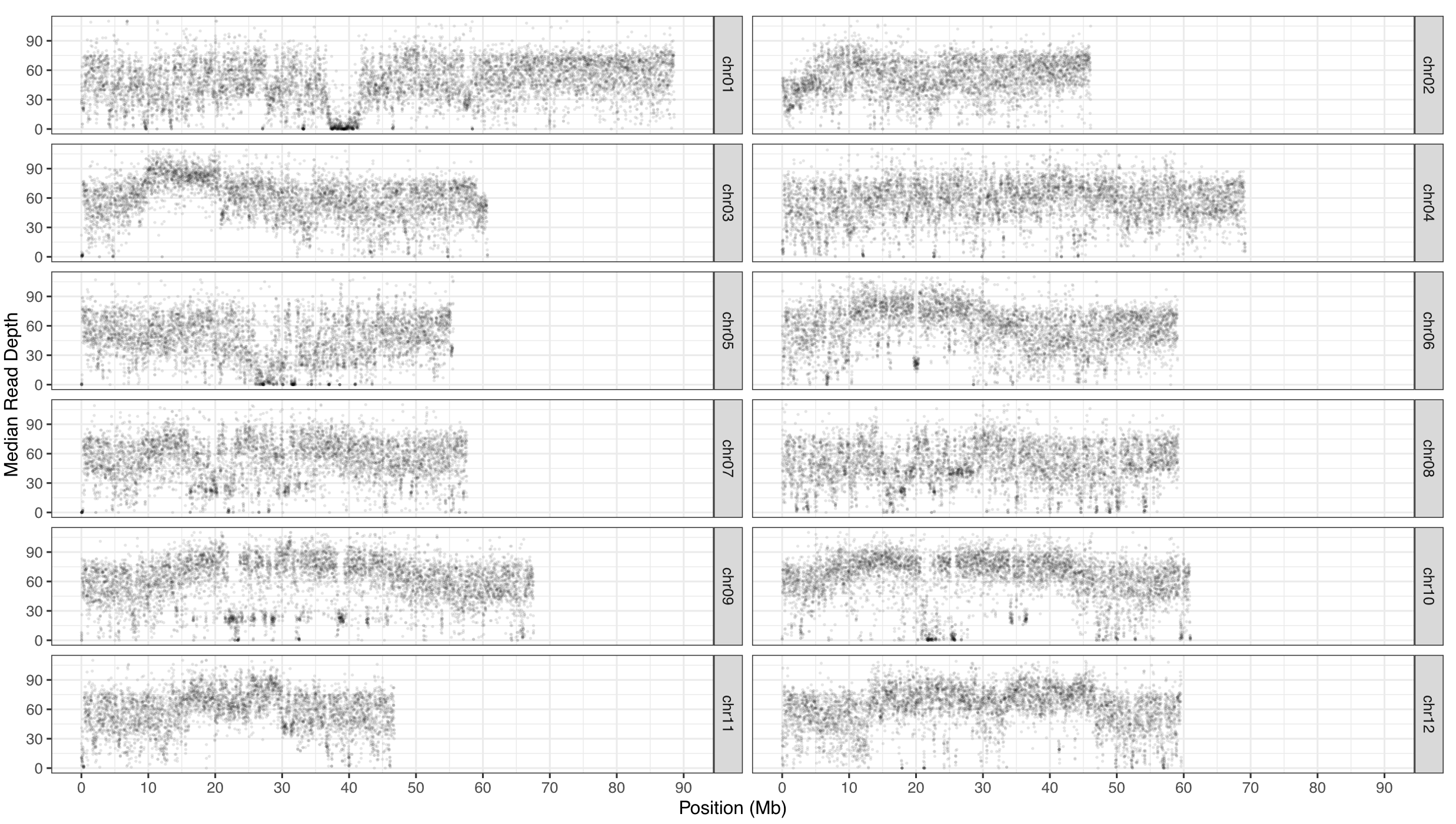 |
| --- |
|  |

**Supplemental Figure 2**. (Continues on pages 3 and 4. Legend on page 4)

TXNS296 and TXNS278

|  |
| --- |
| 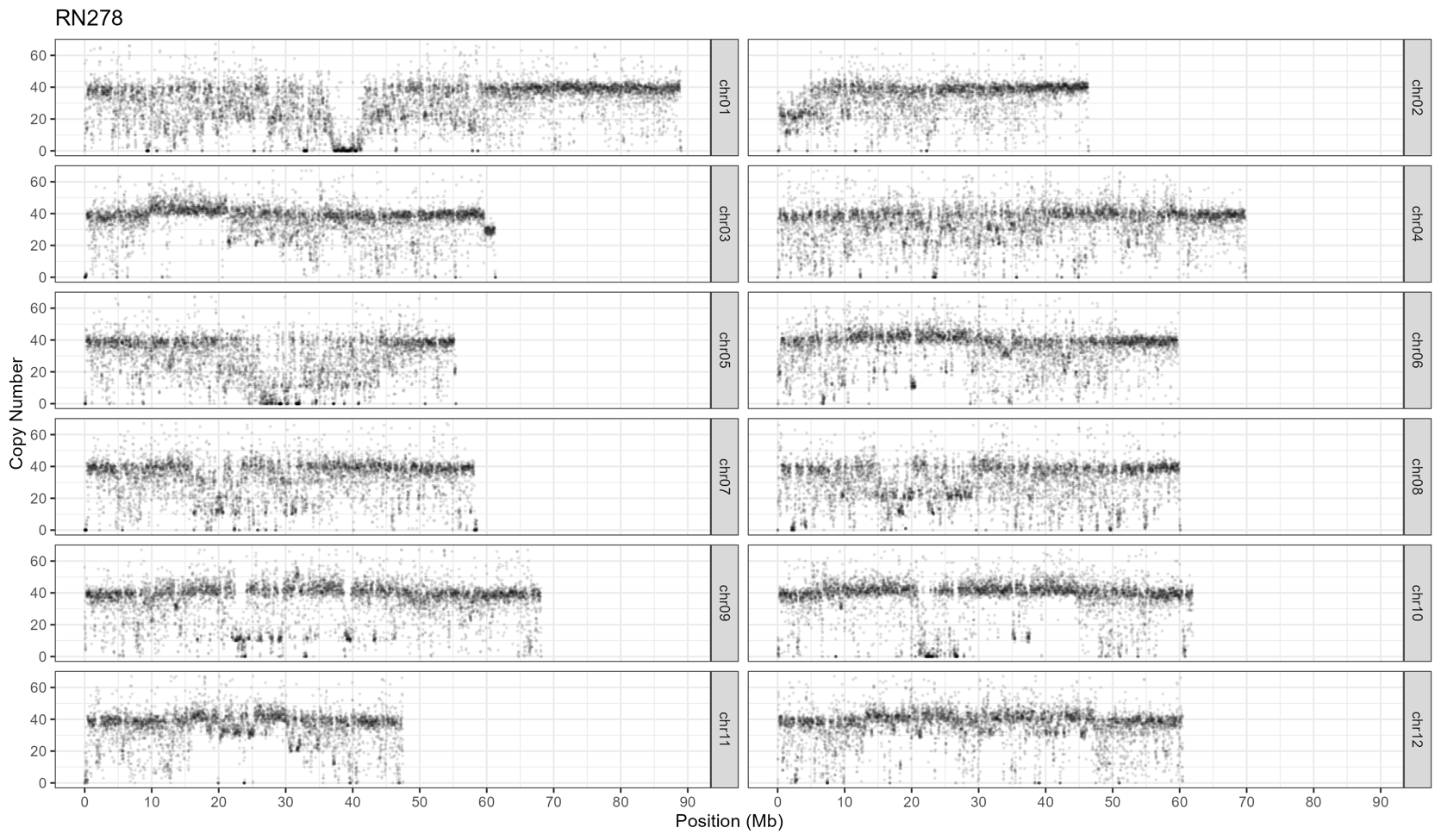 |

**Supplemental Figure 2**. (Continues, legend on page 4)

TXNS223 and TXNS112

| 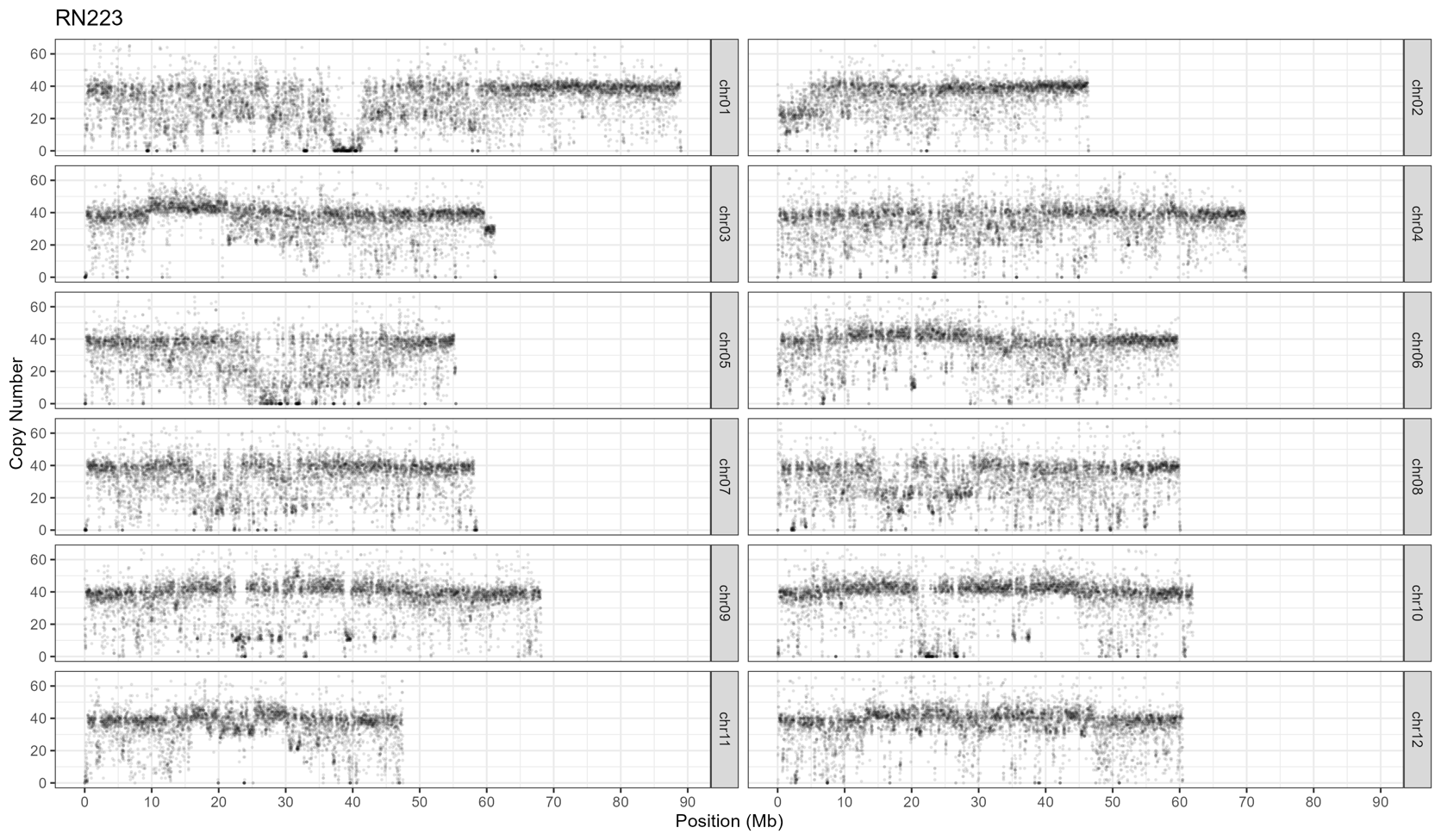 |
| --- |
| 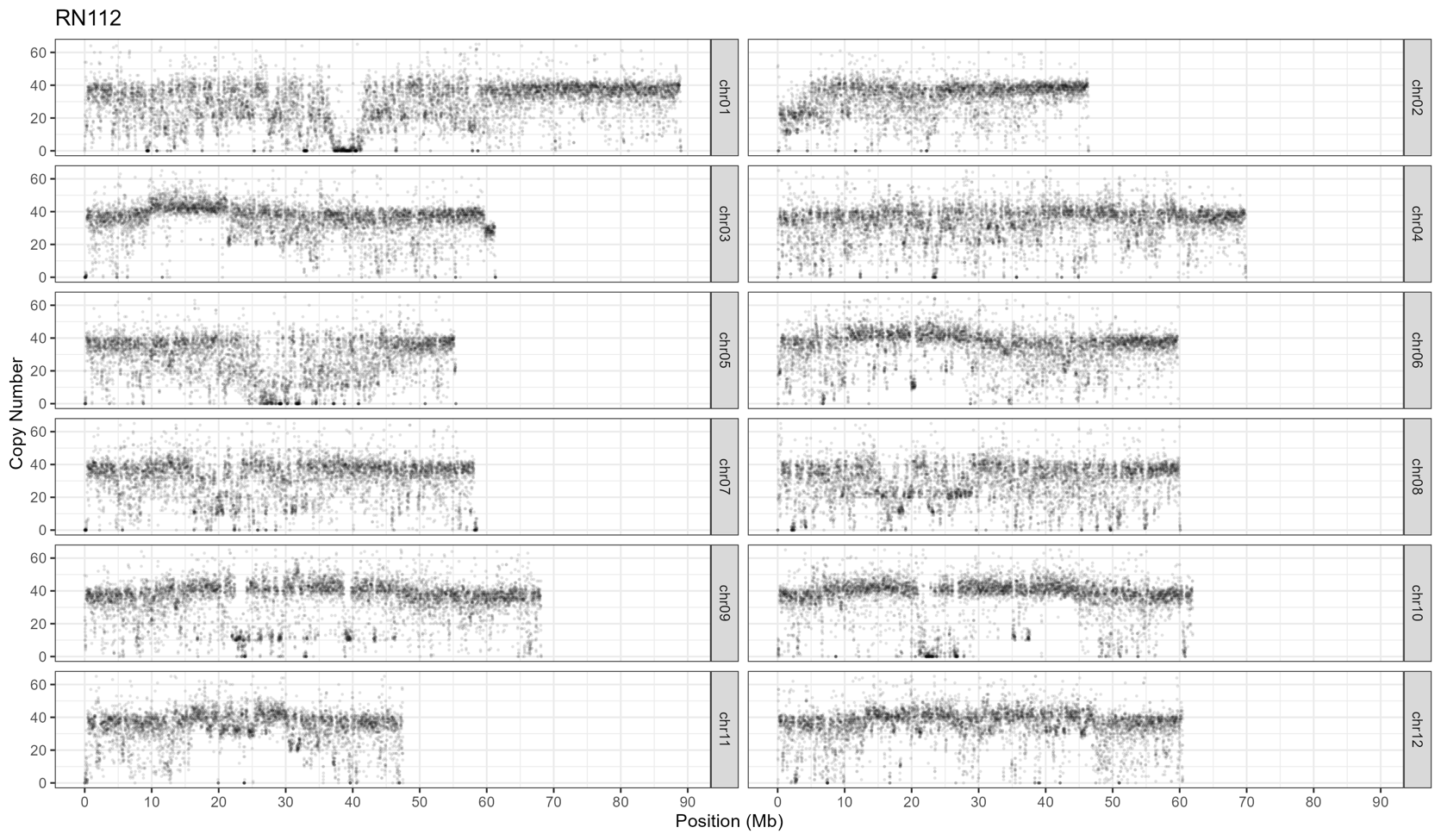 |

**Supplemental Figure 2**. Dosage plots of Russet Norkotah and its Texas TXNS# strains. For dosage analysis, read depth is binned in 100kb intervals and plotted along each chromosomal axis. In the euchromatic chromosomal arms, the Solacap reads have average depth of 60. The TXNS# reads of 40. The highly variable regions are typically centromeric and pericentromeric.

| Clone | Left view | Right view |
| --- | --- | --- |
| RN | 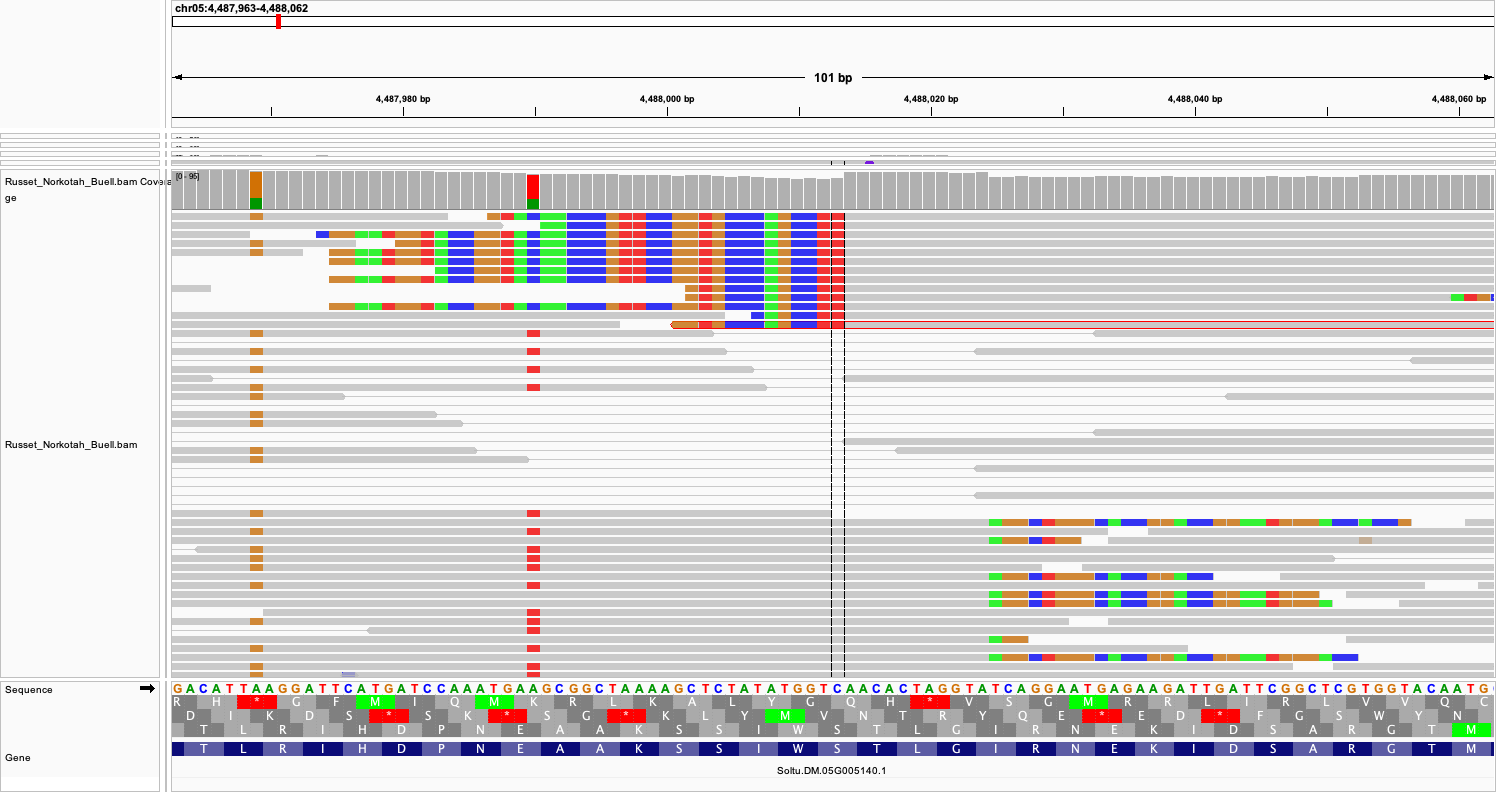 | 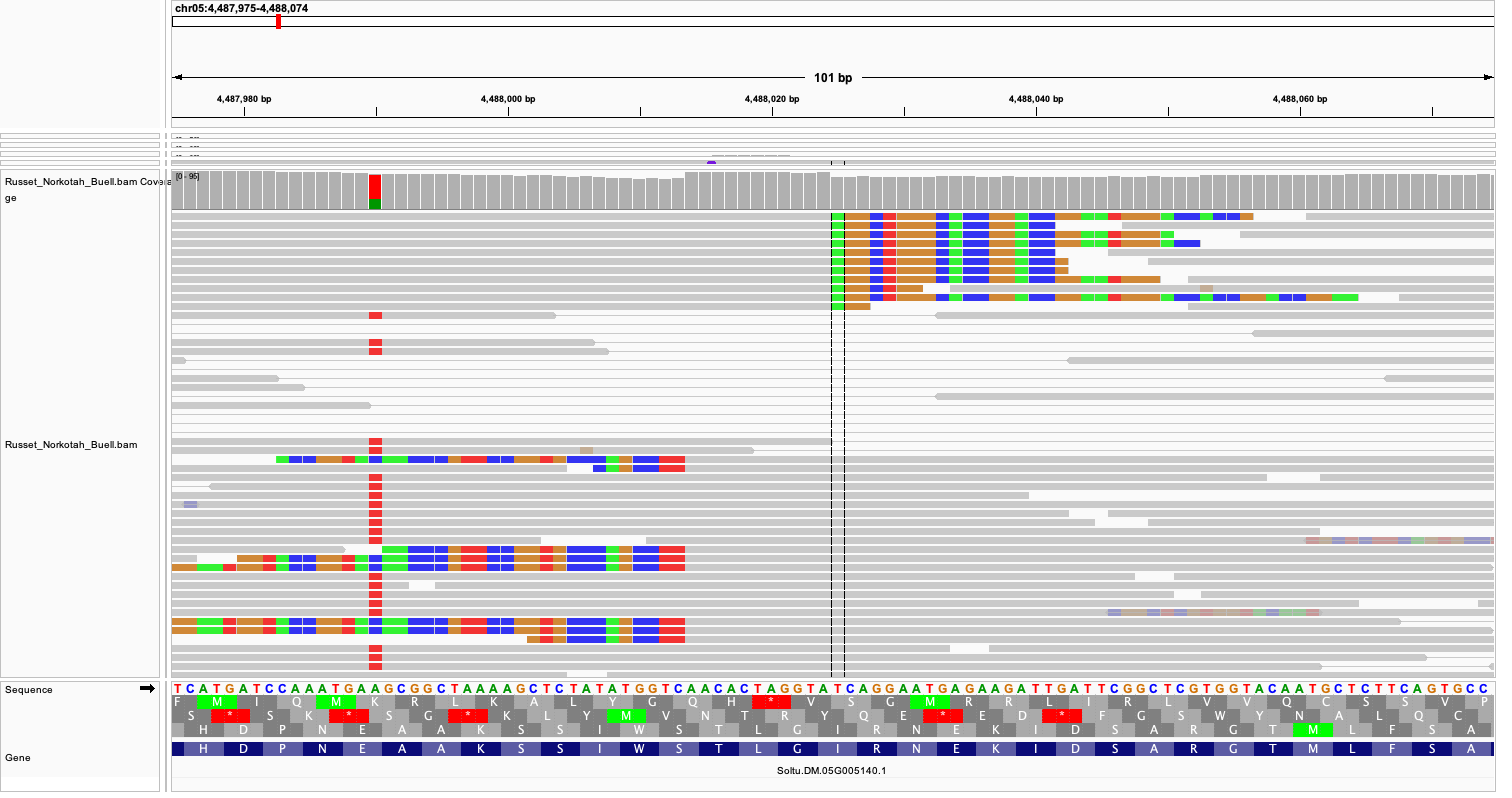 |
| TXNS112 | 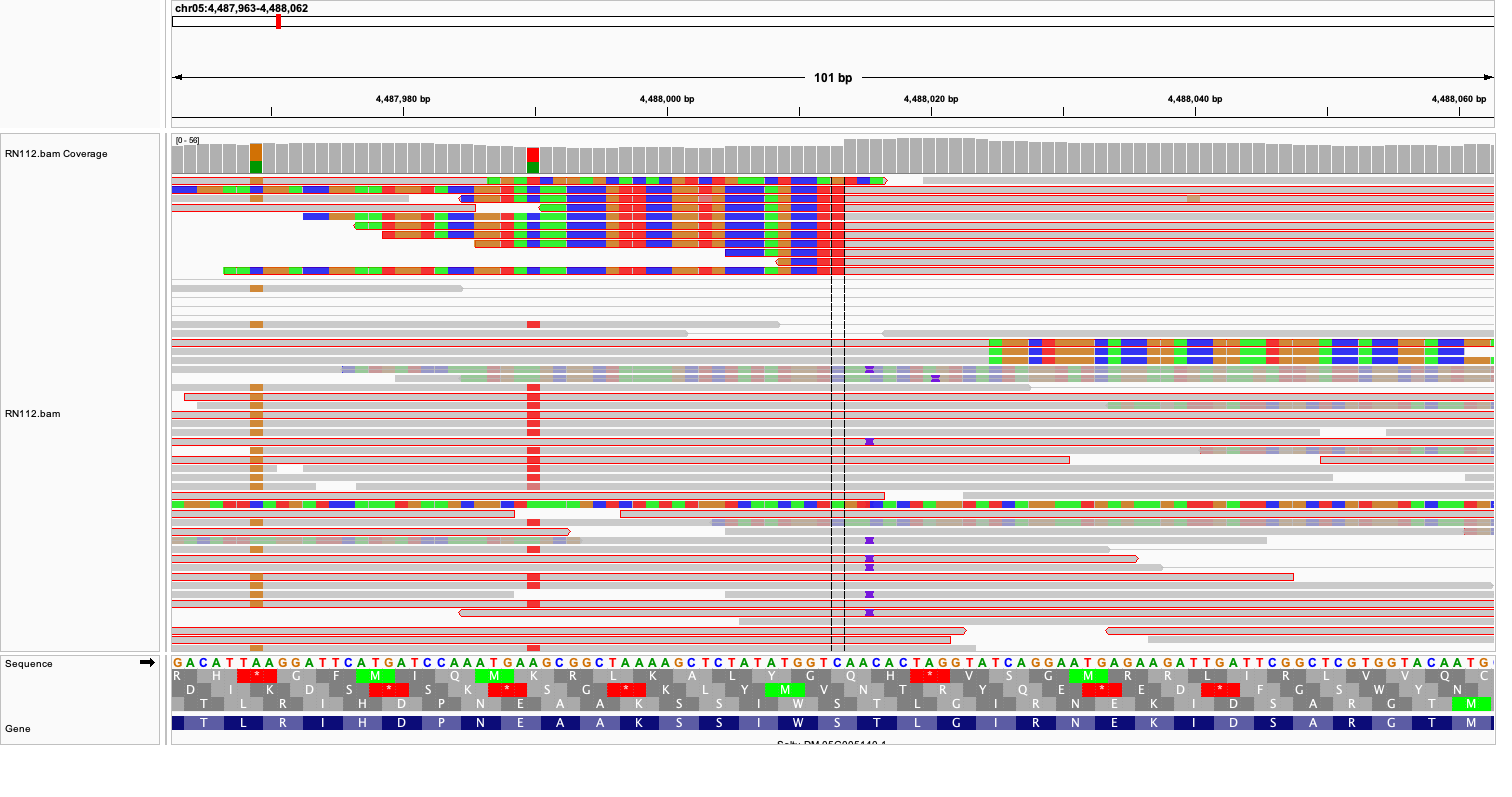 | 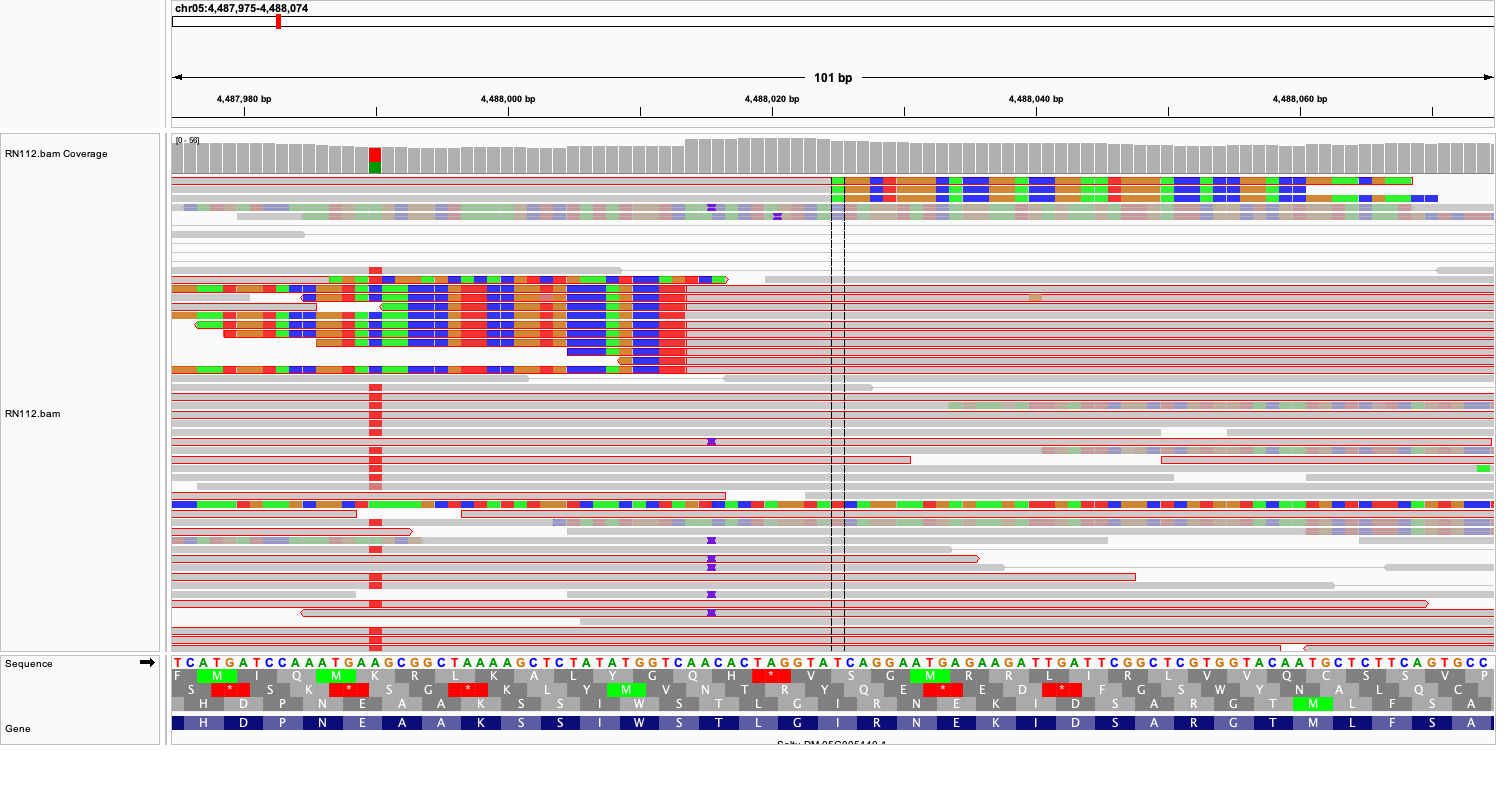 |
| TXNS223 | 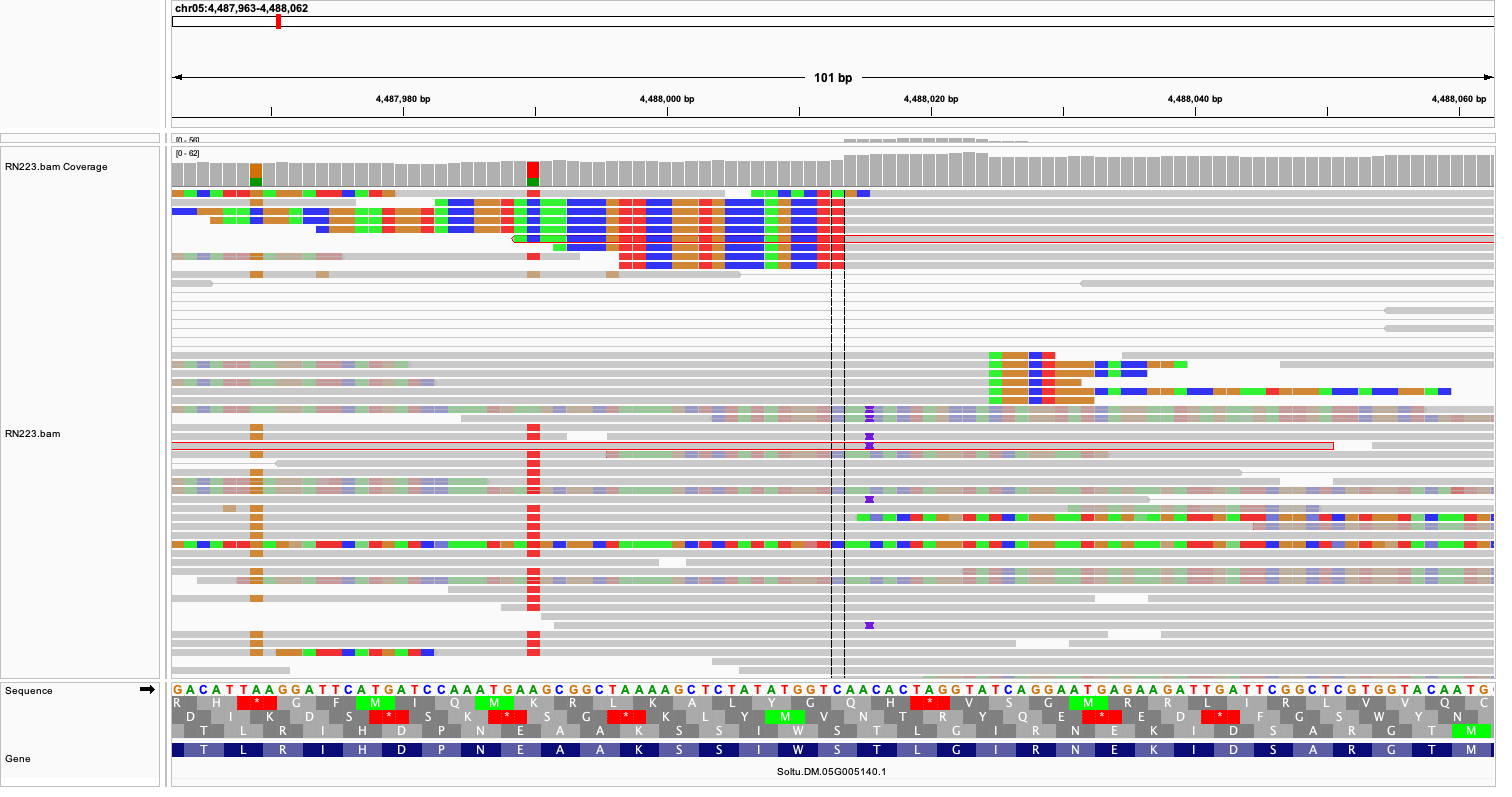 | 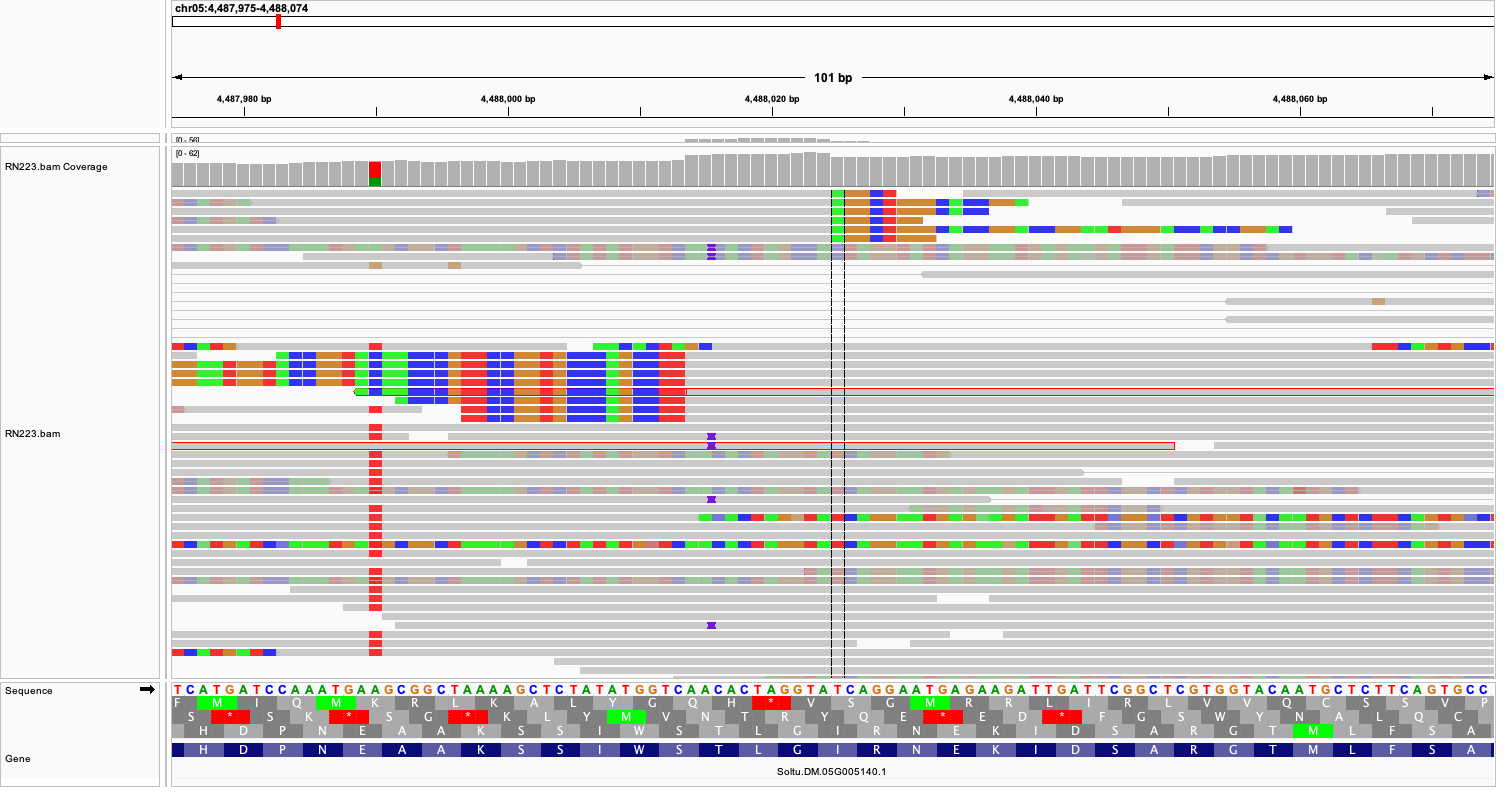 |
| TXNS278 | 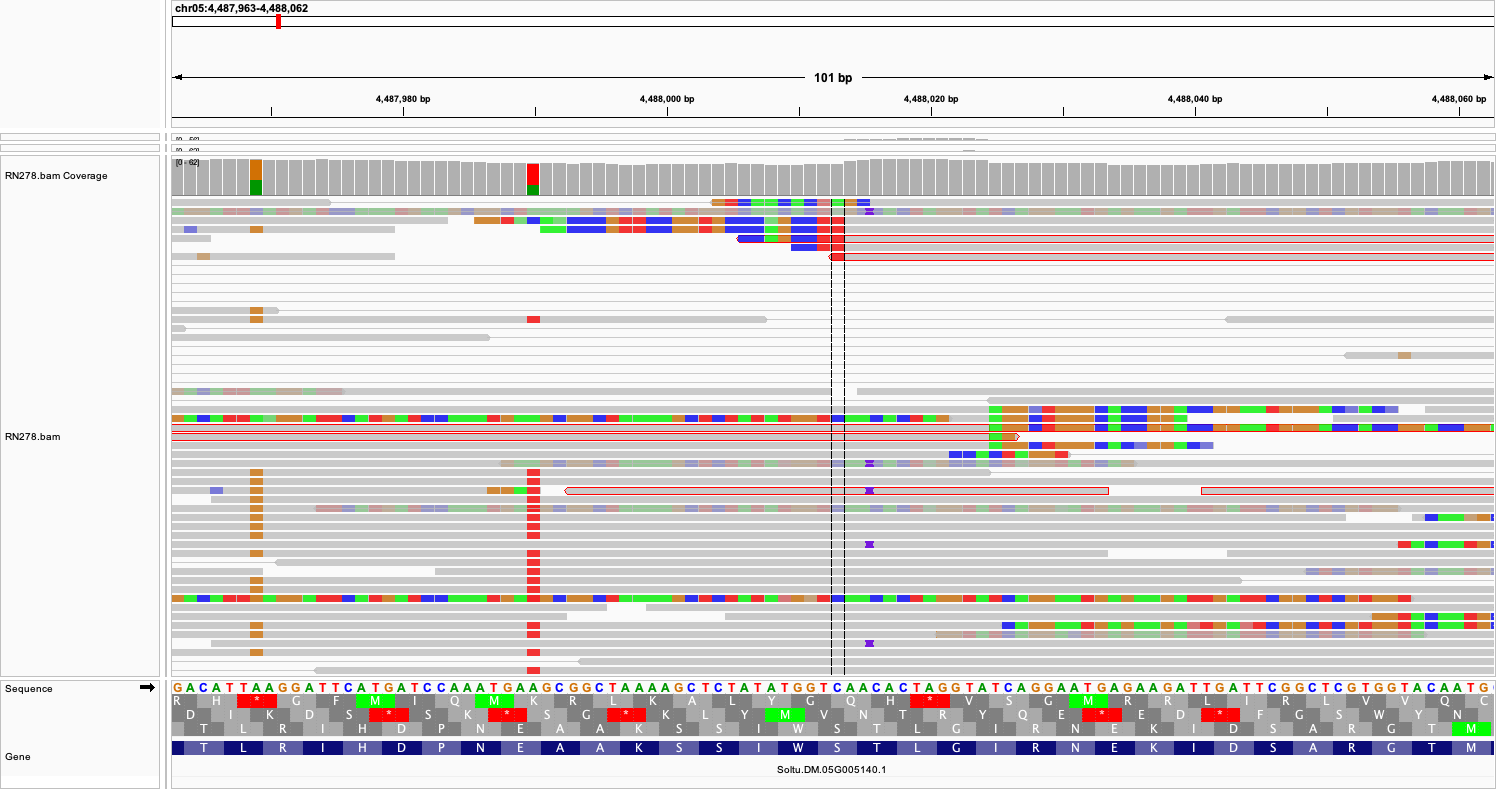 | 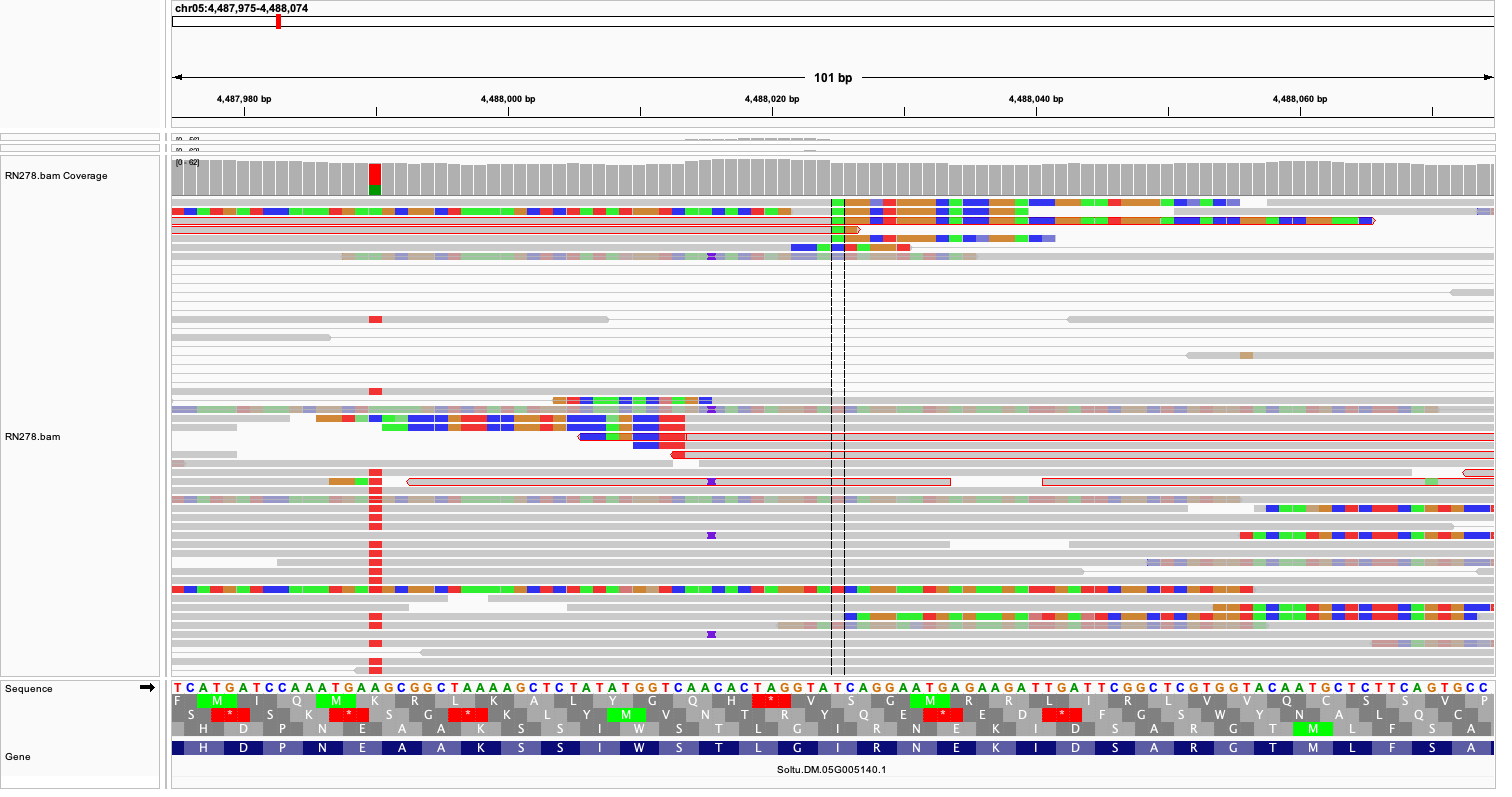 |
| TXNS296 | 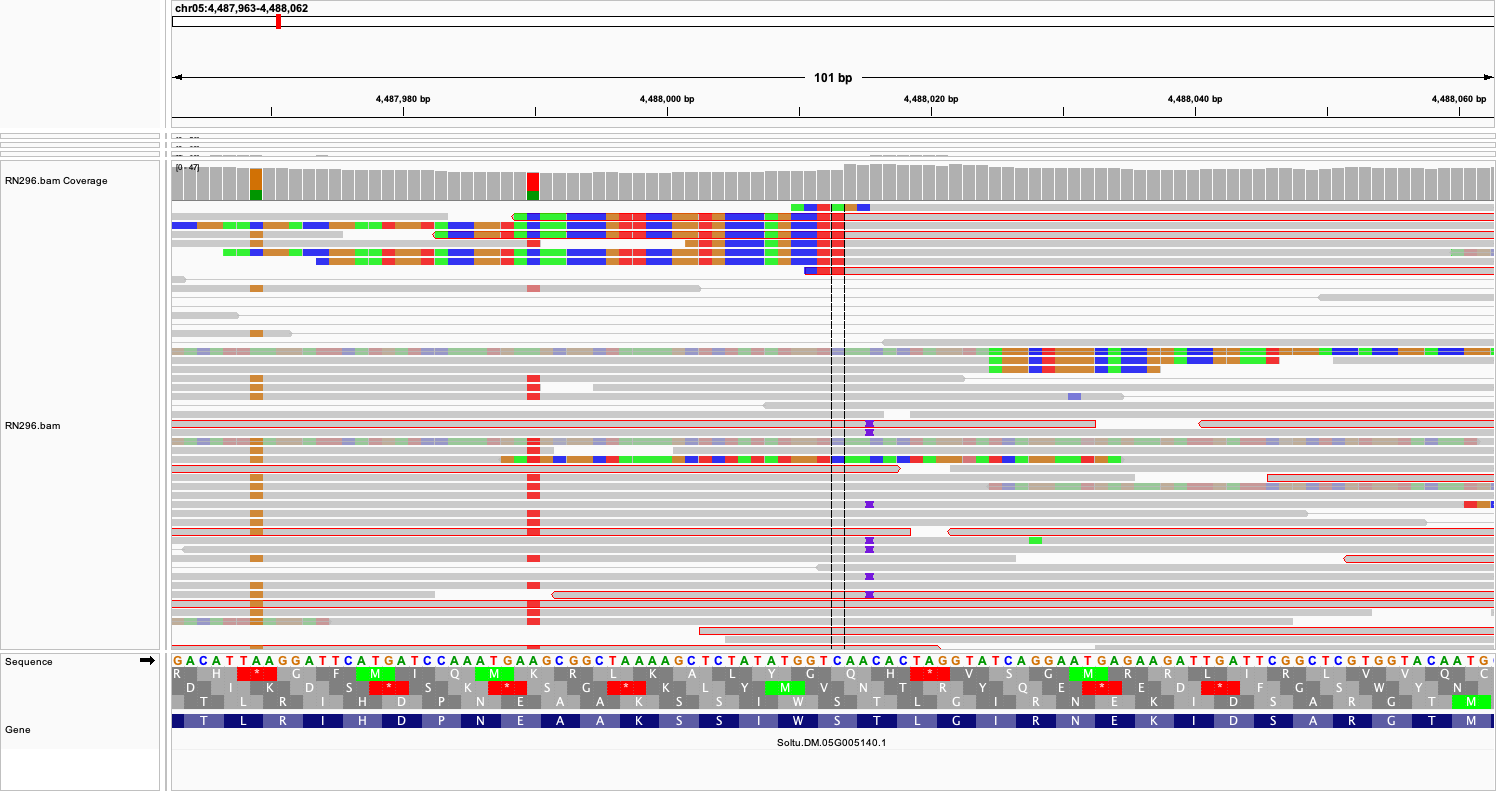 | 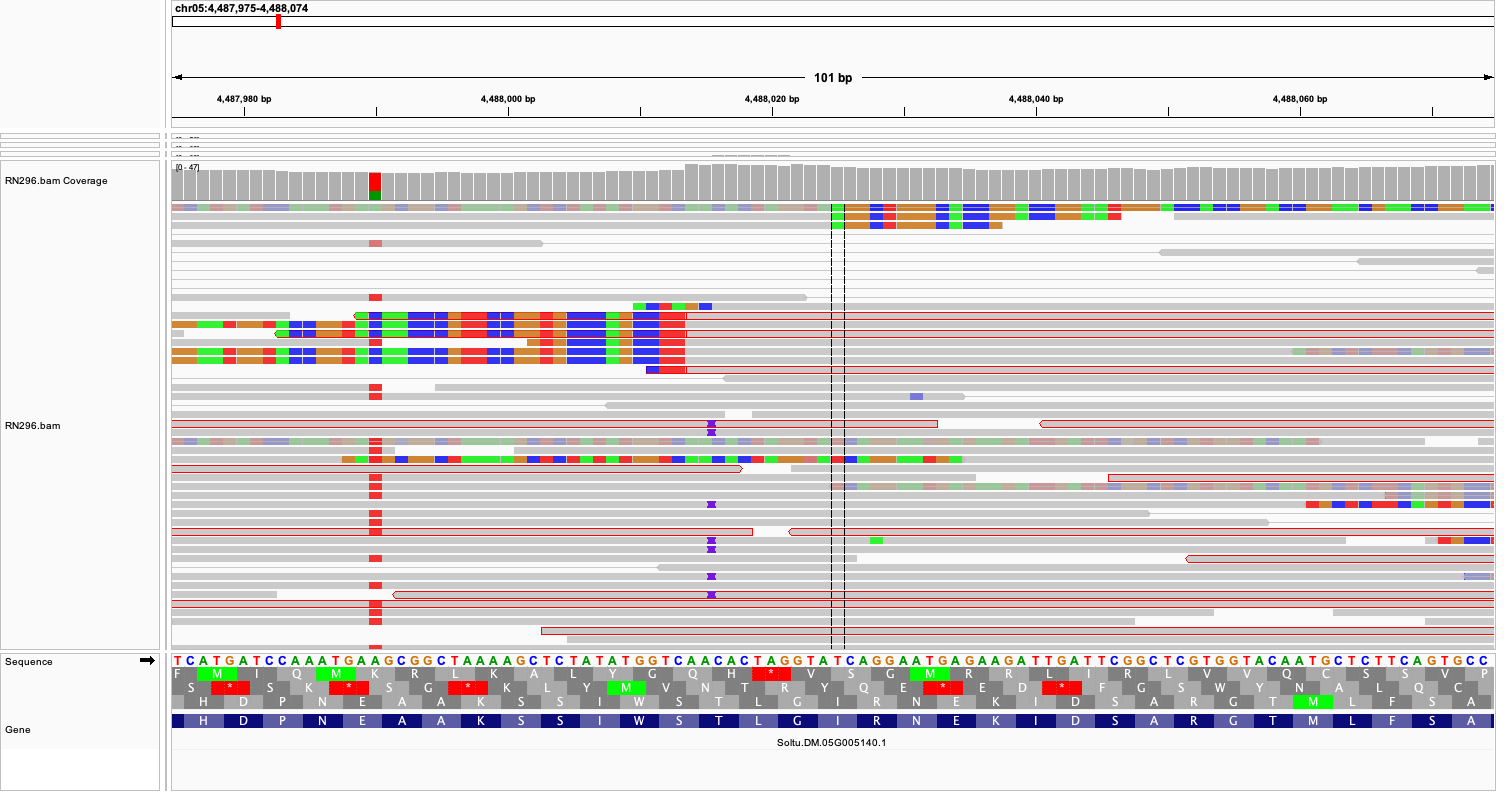 |

**Supplemental Figure 3**. Short genomic read alignment to the second exons of the *CDF1* locus.

| Sample | Integrative Genome Viewer alignment of sequence reads |
| --- | --- |
| TXNS278 Leaf  Russet Norkotah Leaf | 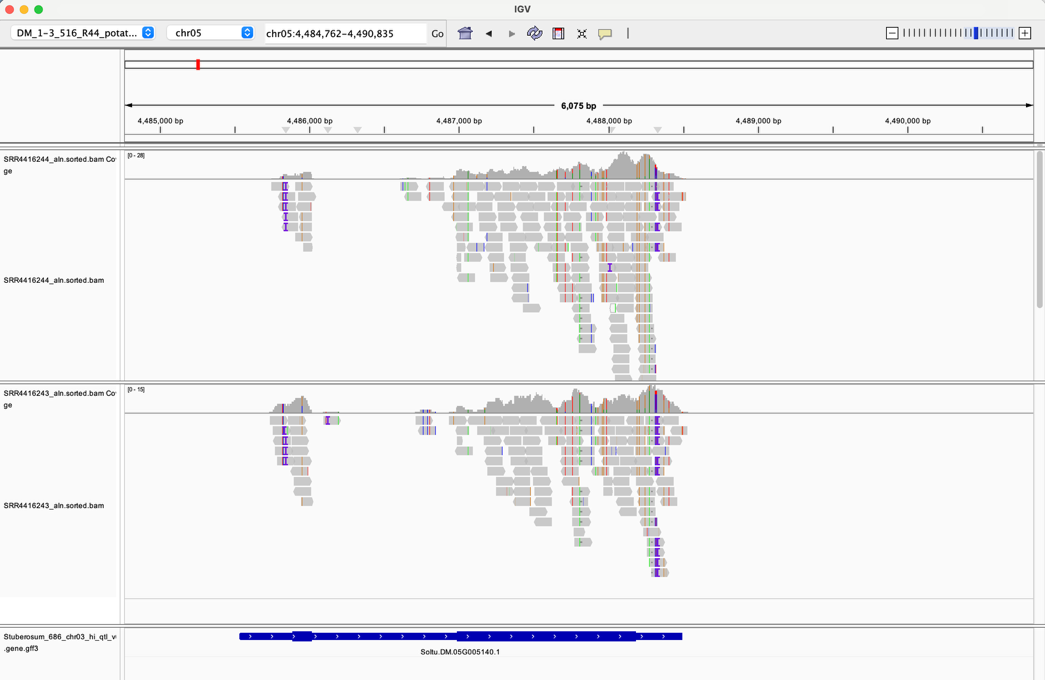 |
| TXNS278 Leaf  Russet Norkotah Leaf | 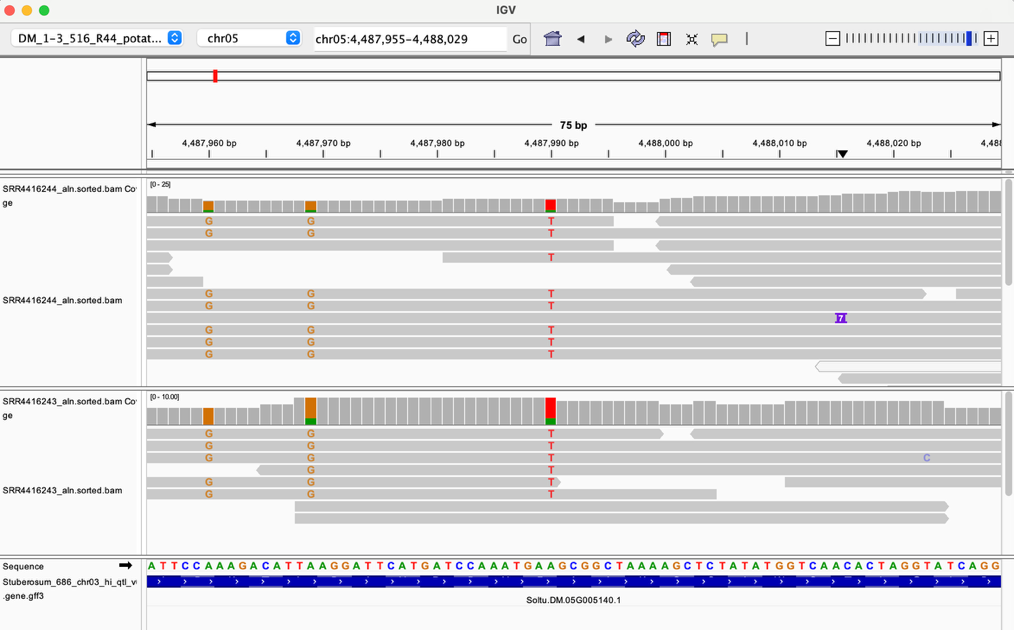 |
|  |  |

**Supplemental Figure 4**. Short RNAseq read alignment to the *CDF1* locus (top) and to the second exon’s region (bottom) containing the 7bp duplication (position 4,888,016 of chr.5) of allele *CDF1.2*. Most reads diplay the G, G, and T SNPs and correspond to alleles *CDF1.1.*
