# Supplementary material for: Clonal strains of the fresh-market potato cultivar Russet Norkotah changed the domestication gene CDF1
Source: Plant Physiol. 2025 Aug 13;198(4):kiaf321. doi: 10.1093/plphys/kiaf321 (PMC12344491; doi:10.1093/plphys/kiaf321)
Supplement: kiaf321_Supplementary_Data [file kiaf321_supplementary_data.zip › Amundson_supplemental_tables.docx]

**Clonal strains of the fresh-market potato cultivar Russet Norkotah changed the domestication gene CDF1**

Kirk Amundson^1,2^ , M. Isabel Vales^3^, Isabelle J. DeMarco^1^, Weier Guo^1^, Isabelle M. Henry^1^, and Luca Comai^1^,*

^1^Department of Plant Biology and Genome Center, University of California Davis, Davis, CA 95616

^2^Current address: Department of Biology, University of Massachusetts Amherst, Amherst, MA 01003

^3^Department of Horticultural Sciences, Texas A&M University, College Station, TX 77843

# Supplementary information

| **chrom** | **pos** | ***CDF1***  **exon** | **Ref** | **Alt** | **Ref**  **codon** | **Alt**  **codon** | **Ref**  **aa** | **Alt**  **aa** | **Effect** | **Alt phase with hAT TE** | **Alt allele**  **dosage** | **RN**  **hap1,2** | **RN_**  **hap3,4** | **TXNS# *CDF1.2*** |
| --- | --- | --- | --- | --- | --- | --- | --- | --- | --- | --- | --- | --- | --- | --- |
| chr05 | 4485952 | 1 | C | G | ACC | AGC | Thr | Ser | missense | unknown | 2 |  |  |  |
| chr05 | 4487060 | 2 | G | A | TCG | TCA | Ser | Ser | silent | unknown | 2 |  |  |  |
| chr05 | 4487654 | 2 | T | A | GAT | GAA | Asp | Glu | missense | repulsion | 2 | T | A | T |
| chr05 | 4487660 | 2 | A | T | GCA | GCT | Ala | Ala | silent | repulsion | 2 | A | T | A |
| chr05 | 4487711 | 2 | C | T | GGC | GGT | Gly | Gly | silent | repulsion | 2 | C | T | C |
| chr05 | 4487759 | 2 | A | T | ACA | ACT | Thr | Thr | silent | repulsion | 2 | A | T | A |
| chr05 | 4487810 | 2 | T | A | TCT | TCA | Ser | Ser | silent | repulsion | 2 | T | A | T |
| chr05 | 4487819 | 2 | ATC | del | . | . | . | . | out of frame 3bp deletion | repulsion | 2 | ATC | del | ATC |
| chr05 | 4487886 | 2 | A | C | ATT | CTT | Ile | Leu | missense | repulsion | 2 | T | C | T |
| chr05 | 4487916 | 2 | G | A | GCT | ACT | Ala | Thr | missense | repulsion | 2 | G | A | G |
| chr05 | 4487930 | 2 | T | G | AAT | AAG | Asn | Lys | missense | repulsion | 2 | T | G | T |
| chr05 | 4487960 | 2 | A | G | CCA | CCG | Pro | Pro | silent | repulsion | 2 | A | G | A |
| chr05 | 4487969 | 2 | A | G | TTA | TTG | Leu | Leu | silent | repulsion | 2 | A | G | A |
| chr05 | 4487990 | 2 | A | T | GAA | GAT | Glu | Asp | missense | repulsion | 2 | A | T | A |
| chr05 | 4488015 | 2 | A | hAT; hAT excision | . | . | . | . | hAT; TSD | . | 2;1 | hAT | A | hAT excision |

**Supplemental Table 1**. Variants in *CDF1* gene coding sequence. Variants in second exon were dense enough to phase by visualizing short read alignment on the IGV browser. For Russet Norkotah, the four chromosomes display two unique haplotypes. The TE excision in the TXNS# occurred on either hap1 or hap2 and in repulsion with 3bp deletion haplotype.

| **SRA ID** | **Sample** | ***CDF1* allele** | | | |
| --- | --- | --- | --- | --- | --- |
|  |  | ***1.1*** | ***1.2*** | ***1.3*** | ***1.2 or 1.3*** |
| SRR4416243 | RN_leaf_T1 | 6 | 0 | 2 | 0 |
| SRR4416244 | TXNS278_leaf_T1 | 8 | 1 | 0 | 1 |
| SRR4416245 | TXNS278_leaf_T2 | 10 | 0 | 1 | 1 |
| SRR4416246 | RN_root_T1 | 11 | 0 | 1 | 0 |
| SRR4416247 | TXNS278_root_T1 | 8 | 0 | 0 | 1 |
| SRR4416248 | RN_root_T2 | 9 | 0 | 1 | 0 |
| SRR4416249 | TXNS278_root_T2 | 2 | 0 | 0 | 0 |
| SRR6676801 | RN_leaf_T2 | 5 | 0 | 2 | 0 |

**Supplemental Table 2.** Expression of CDF1 alleles. RNAseq reads were inspected between nucleotide 4,487,950 and 4,488,020 for distinguishing polymorphism to determine their allelic origin. *CDF1.2* was identified by the presence of a 7bp duplication at position 4,488,016 of chr.5. Notably, RN tissue is homogeneous for the allele genotype, while it is likely that TXNS278 is a periclinal chimera (see Letter text). This would dilute the *CDF1.2* allele proportionally. RN: Russet Norkotah original clone. TXNS278: clonal variant of RN.
