# Supplementary material for: Clonal strains of the fresh-market potato cultivar Russet Norkotah changed the domestication gene CDF1
Source: Plant Physiol. 2025 Aug 13;198(4):kiaf321. doi: 10.1093/plphys/kiaf321 (PMC12344491; doi:10.1093/plphys/kiaf321)
Supplement: kiaf321_Supplementary_Data [file kiaf321_supplementary_data.zip › Amundson_supplementary_text_07082025.docx]

**Clonal strains of the fresh-market potato cultivar Russet Norkotah changed the domestication gene CDF1**

Kirk R. Amundson^1,2^ , M. Isabel Vales^3^, Isabelle J. DeMarco^1^, Weier Guo^1^, Isabelle M. Henry^1^, and Luca Comai^1^,*

^1^Department of Plant Biology and Genome Center, University of California Davis, Davis, CA 95616

^2^Current address: Department of Biology, University of Massachusetts Amherst, Amherst, MA 01003

^3^Department of Horticultural Sciences, Texas A&M University, College Station, TX 77843

**Supplementary Methods, Data availability, and References**

Plant testing phenotype

Potato field trials followed standard practices described in the Texas A&M Potato Breeding Reports: https://potato.tamu.edu/reports/. The average temperatures and precipitation during the potato growing season have been recorded for all years presented (2007-2018). Precipitation varied year to year, but central pivot irrigation was provided when needed. Plant maturity was assessed by measuring when foliage turns yellow, plants start senescing, and tubers reach full size. The trait was scored using a 1-5 scale, where 1 = Very early (<100 DAP), 2 = Early (100-110 DAP), 3 = Mid-season (111-120 DAP), 4 = Late (121-130 DAP), 5 = Very late (>130 DAP). Plant vigor was evaluated at ~45 days after planting, when plants’ canopies were not yet overlapping, using a subjective visual scale from 1 to 5, where 1 represents poor performance with the lowest vigor and 5 the most vigorous and strongest growth.

Genomic DNA Sequencing and Analysis

Sequence read library construction, read alignment and processing were performed as previously described [(Amundson et al., 2020)](https://paperpile.com/c/yi90ln/QiNk3). Briefly, 750 ng of genomic DNA was used as input for library construction using a KAPA Hyper Prep kit (Roche catalog #KR0961) with half-scale reactions and four cycles of amplification following adapter ligation. All libraries were sequenced on an Illumina NovaSeq 6000 at the University of California, San Francisco. Sequencing reads generated in this report have been deposited at the NCBI Sequence Read Archive under project ID [PRJNA1241303](https://www.ncbi.nlm.nih.gov/sra/PRJNA1241303). Genomic sequence reads from Russet Norkotah leaves [(Pham et al., 2017)](https://paperpile.com/c/yi90ln/eSmKk) were retrieved from NCBI Sequence Read Archive project [PRJNA378971](https://www.ncbi.nlm.nih.gov/bioproject/PRJNA378971). Raw reads were trimmed with Cutadapt 1.15 [(Martin, 2011)](https://paperpile.com/c/yi90ln/V6cfI) to a maximum length of 100 nt, in order to account for differences in read length between Russet Norkotah and the TXNS strains. Trimmed reads were aligned to the DM1-3 v6.1 reference genome [(Pham et al., 2020)](https://paperpile.com/c/yi90ln/sYvqk) using BWA mem (version 0.7.12-r1039) [(Li, 2013)](https://paperpile.com/c/yi90ln/w3sov) with default parameters. PCR duplicates were marked and removed with Picard MarkDuplicates (version 2.18). Paired end reads with mates mapping to different chromosomes were removed using a custom awk script. For overlapping paired-end reads, the overlap region for one of the two mates was soft-clipped using bamUtil clipOverlap [(Jun et al., 2015)](https://paperpile.com/c/yi90ln/4Eohl). RNA-Seq reads of Russet Norkotah and TXNS 278 leaves and roots [(Levy et al., 2018)](https://paperpile.com/c/yi90ln/xeWbG) were retrieved from NCBI Sequence Read Archive project [PRJNA347867](https://www.ncbi.nlm.nih.gov/bioproject/PRJNA347867) and aligned to the DM1-3 v6.1 assembly using hisat2 (version 2.1.0) [(Kim et al., 2019)](https://paperpile.com/c/yi90ln/LQbkg). All read alignments were visualized using the Integrated Genomics Viewer (version 2.16.1) [(Thorvaldsdóttir et al., 2013)](https://paperpile.com/c/yi90ln/ngNza). For haplotype phasing, a minimum read mapping quality threshold of 20 was applied to Russet Norkotah and all TXNS clones. Mappability was calculated across the CDF1 locus using GenMap (version 1.2.0) [(Pockrandt et al., 2020)](https://paperpile.com/c/yi90ln/GPn2e), setting k=100 and conducting a parameter sweep of *e* from 0 to 4 in increments of 1. At all tested mismatch rates, mappability scores across the entire CDF1 locus were 1 (not shown), ruling out complicating effects of read mappability. Raw variants were called using freebayes (version 1.3.4) (Garrison and Marth, 2012) and annotated using snpEff (Cingolani 2012). We removed sites with a variant quality score less than 20, with ≤8x read coverage in Russet Norkotah or any TXNS strain, or with total depth across all samples exceeding 450 from further consideration. To test whether the TXNS strains were clones, genotypes were variants at 1,640 SNP loci, and identity-by-state distances were compared against a consolidated panel of 1,181 potato cultivars and breeding lines as previously described (Amundson et al. 2023). To identify candidate mutations that arose specifically in the TXNS strains, we retained sites for which the reference or alternate allele-specific read coverage was exactly zero in Russet Norkotah and three or more in at least one TXNS mutant. For mutations shared among all four TXNS strains, the minimum mutant allele coverage was left at 3x. To identify mutations specific to one TXNS strain, we used a more stringent mutant allele coverage cutoff of ≥7x and required strict absence of the mutant allele in the other strains. Using these thresholds of ≥7x mutant allele coverage in focal samples and strict absence of a mutant allele in non-focal samples, we identified all mutations shared by groups of 2 or 3 TXNS strains.

**References Cited**

Amundson KR, Ordoñez B, Santayana M, Tan EH, Henry IM, Mihovilovich E, Bonierbale M, Comai L (2020) Genomic outcomes of haploid induction crosses in potato (Solanum tuberosum L.). Genetics 214: 369–380

Amundson KR, Marimuthu MPA, Nguyen O, Sarika K, DeMarco IJ, Phan A, Henry IM, Comai L (2023) Differential mutation rates in plant meristematic layers. bioRxiv 2023.09.25.559363

Caraza-Harter MV, Endelman JB (2022) The genetic architectures of vine and skin maturity in tetraploid potato. Züchter Genet Breed Res 135: 2943–2951

Hoopes G, Meng X, Hamilton JP, Achakkagari SR, de Alves Freitas Guesdes F, Bolger ME, Coombs JJ, Esselink D, Kaiser NR, Kodde L, et al (2022) Phased, chromosome-scale genome assemblies of tetraploid potato reveals a complex genome, transcriptome, and predicted proteome landscape underpinning genetic diversity. Mol Plant. doi: 10.1016/j.molp.2022.01.003

Jun G, Wing MK, Abecasis GR, Kang HM (2015) An efficient and scalable analysis framework for variant extraction and refinement from population-scale DNA sequence data. Genome Res 25: 918–925

Kim D, Paggi JM, Park C, Bennett C, Salzberg SL (2019) Graph-based genome alignment and genotyping with HISAT2 and HISAT-genotype. Nat Biotechnol 37: 907–915

Levy J, Tamborindeguy C, Athrey G, Scheuring DC, Koym JW, Miller JC Jr (2018) Transcriptome of Russet Norkotah and its clonal selection, TXNS278. BMC Res Notes. doi: 10.1186/s13104-018-3254-4

Li H (2013) Aligning sequence reads, clone sequences and assembly contigs with BWA-MEM. arXiv [q-bio.GN]

Martin M (2011) Cutadapt removes adapter sequences from high-throughput sequencing reads. EMBnet J 17: 10

Pham GM, Hamilton JP, Wood JC, Burke JT, Zhao H, Vaillancourt B, Ou S, Jiang J, Buell CR (2020) Construction of a chromosome-scale long-read reference genome assembly for potato. Gigascience. doi: 10.1093/gigascience/giaa100

Pham GM, Newton L, Wiegert-Rininger K, Vaillancourt B, Douches DS, Buell CR (2017) Extensive genome heterogeneity leads to preferential allele expression and copy number‐dependent expression in cultivated potato. Plant J 92: 624–637

Pockrandt C, Alzamel M, Iliopoulos CS, Reinert K (2020) GenMap: ultra-fast computation of genome mappability. Bioinformatics 36: 3687–3692

Thorvaldsdóttir H, Robinson JT, Mesirov JP (2013) Integrative Genomics Viewer (IGV): high-performance genomics data visualization and exploration. Brief Bioinform 14: 178–192
