# Supplementary material for: Clonal strains of the fresh-market potato cultivar Russet Norkotah changed the domestication gene CDF1
Source: Plant Physiol. 2025 Aug 13;198(4):kiaf321. doi: 10.1093/plphys/kiaf321 (PMC12344491; doi:10.1093/plphys/kiaf321)
Supplement: kiaf321_Supplementary_Data [file kiaf321_supplementary_data.zip › Amundson_Suppl_Datasets.docx]

**Clonal strains of the fresh-market potato cultivar Russet Norkotah changed the domestication gene CDF1**

Kirk Amundson^1,2^ , M. Isabel Vales^3^, Isabelle J. DeMarco^1^, Weier Guo^1^, Isabelle M. Henry^1^, and Luca Comai^1^,*

^1^Department of Plant Biology and Genome Center, University of California Davis, Davis, CA 95616

^2^Current address: Department of Biology, University of Massachusetts Amherst, Amherst, MA 01003

^3^Department of Horticultural Sciences, Texas A&M University, College Station, TX 77843

# Supplemental Datasets

**[LINK FOR REVIEW: https://figshare.com/s/3ba667841d1129dc4092]**

**Supplemental Table 3**. RN high-confidence mutations (RN_high_confidence_mutations_v2.tsv). Stringently filtered of mutations in RN and its clones. 10.6084/m9.figshare.29298572.

**Supplemental Table 4**. RN lower stringency mutations (RN_rough_mutations_v2.tsv). Standardly filtered mutations in RN and its clones. 10.6084/m9.figshare.29298572

**Supplemental Table 5**. SNPeff analysis of mutations (filt2_q20_rn_calls_v2_snpeff.vcf.gz). 10.6084/m9.figshare.29298572
